# Demonstration of the histopathological and immunohistochemical effects of a novel hemostatic agent, ankaferd blood stopper, on vascular tissue in a rat aortic bleeding model

**DOI:** 10.1186/1749-8090-5-110

**Published:** 2010-11-14

**Authors:** Ozer Kandemir, Mustafa Buyukates, Nilufer Onak Kandemir, Erol Aktunc, Aylin Ege Gul, Sanser Gul, S Akin Turan

**Affiliations:** 1 Department of Cardiovascular Surgery, Zonguldak Karaelmas University, Zonguldak, Turkey; 2 Department of Pathology, Zonguldak Karaelmas University, Zonguldak, Turkey; 3 Department of Family Medicine, Zonguldak Karaelmas University, Zonguldak, Turkey; 4 Department of Pathology, Dr.Lutfu Kirdar Research and Training Hospital, Istanbul, Turkey; 5 Department of Neurosurgery, Zonguldak Karaelmas University, Zonguldak, Turkey

## Abstract

**Background:**

Ankaferd Blood Stopper^® ^(ABS) is a folkloric medicinal plant extract used as a hemostatic agent in traditional Turkish medicine. This experimental study investigated the histopathological and immunohistochemical effects of ABS on vascular tissue in a rat model of aortic bleeding.

**Methods:**

Four groups of 11 Wistar albino rats were used. The abdominal aortas of the rats were wounded; an ABS-soaked tampon was applied to rats in Groups 1 and 3, and a plain gauze tampon was applied to rats in Groups 2 and 4 until the bleeding stopped. The bleeding time was recorded. Immediately following sacrificing, the arteriotomy sites from Groups 1 and 2 were removed. The abdominal incisions in Groups 3 and 4 were closed following hemostasis. On Day 7 of the study, Group 3 and 4 rats were sacrificed and the abdominal aorta arteriotomy sites were removed for histopathological and immunohistochemical evaluation.

**Results:**

The mean bleeding time in 15 animals in Groups 2 and 4 was 4.9 ± 0.6 s, and in 22 animals in Groups 1 and 3 was 3.1 ± 0.6 s. Distal aortic occlusion was not observed on either Day 1 or 7 in any group. Significantly more widespread and dense endothelial nitric oxide synthase (eNOS) staining was observed in Group 1 animals than Group 2. On Days 1 and 7 after application of ABS, histopathological changes, consisting of necrosis, inflammation, and endothelial cell loss, in the rat abdominal aortas did not differ between Groups 1 and 2. The basophilic discoloration in the ABS group on the operation day was a result of a foreign body reaction and hemosiderin-loaded histiocyte accumulation, which occurred on Day 7.

**Conclusions:**

In this study, hemostasis was successfully achieved with ABS in rat abdominal aortas. No histopathological change was found in the rat abdominal aortas between the ABS and control groups on Days 1 and 7. Further studies on the long-term effects of foreign body reactions and hemosiderin-loaded histiocyte accumulation are required.

## Background

Impaired tissue integrity and uncontrollable hemorrhage are important causes of morbidity and mortality, especially in the presence of coagulopathies [1]. Various hemostatic agents have been developed to achieve sufficient hemostasis [2,3]. In cardiovascular surgery, bleeding from anastomosis sites is usually controlled with pressure or additional suturing techniques. Occasionally, these techniques may be insufficient, requiring tissue adhesives as supportive agents [4,5]. Additionally, blind suturing for blood oozing from sutured vascular segments may impair the quality of anastomosis.

To preserve the quality of anastomosis, adjuvant topical hemostatic agents are favored in cardiac and vascular surgery. However, topical hemostatic agents may have disadvantages, such as limited efficacy, limited availability, limited vascular biological compatibility, expensiveness, and risk of infection as a result of the requirement for human blood for commercial production of collagen, thrombin, and prothrombin [6]. Surgeons should also be trained in the use of hemostatic agents, such as fibrin glues.

Ankaferd Blood Stopper^® ^(ABS) is a folkloric medicinal plant extract used as a hemostatic agent in traditional Turkish medicine [7]. The use of this product was approved by the Ministry of Health, Turkey, on October 26, 2007.

In a recent literature search, we found no study on the histopathological and immunohistochemical effects of ABS on vascular tissue. In this experimental study, we investigated the effects of ABS on vascular tissue in a rat model of aortic bleeding.

## Methods

Wistar albino (WA) rats were used to demonstrate the vascular histopathological and immunohistochemical changes following the application of ABS (Trend Teknoloji Ilac AS, Istanbul, Turkey) on the abdominal aorta.

The experimental procedure was approved by the Committee for Animal Research at Zonguldak Karaelmas University School of Medicine. All animal studies conformed with the animal experiment guidelines of the Committee for Humane Care. All animals received care in compliance with the "Principles of Laboratory Animal Care" formulated by the National Society for Medical Reseacrh and "Guide for the Care and the Use of Laboratory Animlas" prepared by the US Natinoal Academy of Sciences and published by the US Natinoal Institute of Health (NIH Publications, No:80-23)

### Animals

Male adult WA rats (Zonguldak Karaelmas University Laboratories, Zonguldak, Turkey), weighing 250-300 g, were maintained on a 12/12-h light/dark cycle and fed *ad libitum*. All animals were housed in individual cages in a temperature-controlled environment (20 ± 2°C). The rats were randomly assigned into ABS and control groups.

### Surgical procedure

All animals were anesthetized with intramuscularly administered ketamine hydrochloride (75 mg/kg). Postoperative analgesia was provided by 1-2 mg/mL paracetamol added to the drinking water. The abdominal aorta was accessed surgically by a midline abdominal incision using sterile technique. The retroperitoneum was explored and the aorta was exposed. The abdominal aorta was wounded just proximal to the iliac bifurcation using an iris blade. ABS solution (1 mL) in a glass vial was poured on a gauze tampon through a syringe. Either an ABS-soaked or plain gauze tampon was applied to the vascular wound, and the bleeding time was recorded. In case of insufficient hemostasis using either of the tampons, an 8/0 Prolene suture (Prodek, Sutures Ltd, UK) was used to provide hemostasis. Aortic sampling was performed in all rats to search for immediate and Day-7 postoperative histopathological changes in vascular tissues as a result of ABS.

### Bleeding assay

The duration of bleeding was measured using a chronometer and defined as the time from wounding until the time bleeding stopped.

### Animal groups

The abdominal aortas of the animals were wounded. ABS-soaked tampons were applied in Group 1 (*n *= 11), and plain gauze tampons were applied in Group 2 (*n *= 11) until the bleeding stopped. All of these animals were sacrificed by cervical dislocation on the operation day.

The abdominal aortas of Groups 3 (*n *= 11) and 4 (*n *= 11) were wounded, and hemostasis was provided with ABS-soaked tampons in Group 3 and plain gauze tampons in Group 4. The abdominal incisions in these two groups were closed following hemostasis. They were kept alive for 7 days and fed *ad libitum*. On Day 7 after the operation, all Group 3 and 4 animals were sacrificed by cervical dislocation. Immediately following sacrifice, the arteriotomy sites from all 44 animals were removed en bloc with a safety margin of 1 mm of untouched aortic vascular tissue both distal and proximal to the wound site.

### Histopathological procedure

All specimens were fixed in 10% phosphate-buffered formaldehyde solution for 24 h at room temperature. Each specimen was cut into three sections: the proximal, intact part of the aorta, the wounded part of the aorta, and the distal, intact part of the aorta.

Following the dehydration process using graded ethanols, specimens were embedded in paraffin blocks and cut into 5-μm-thick sections to be mounted on glass slides. Sections were then deparaffinized with xylene and counterstained with hematoxylin and eosin (H&E), iron blue, and Elastic van Gieson (EVG). EVG staining was performed to identify the external and internal elastic lamina. Iron blue staining was performed to identify hemosiderin. All of the sections were examined in 10 random fields at ×40 magnification using a light microscope. Blinded light microscopic examinations were performed by two of the coauthors (NOK, AEG).

### Histopathological grading of the specimens

Light microscopic findings were graded semi-quantitatively from 0 (no histopathological change) to +3 (severe histopathological change). This histopathological grading was performed for vascular (endothelial cell loss, inflammatory reaction, medial necrosis, fibrin plug formation, and erythrocyte aggregation) and perivascular (inflammatory reaction, hemosiderin-loaded histiocytes, and granulation tissue formation) connective tissue reactions in the specimens.

### Immunohistochemical procedure

In immunohistochemical surveys, anti-CD31 was used to monitor vascular endothelial cells, and anti-eNOS antibodies were used to determine eNOS expression of endothelial cells. For immunohistochemical studies, immunostaining was performed according to the avidin-biotin-peroxidase (BSA-DAB) complex technique. Paraffin sections were collected on slides, deparaffinized, and dehydrated. Endogenous peroxidase activity was blocked using a 3% hydrogen peroxide solution for 10 min. To enhance staining, heat-induced epitope retrieval was performed. Primary antibodies against CD31 (rabbit monoclonal, JC70, Dako, Copenhagen, Denmark) and endothelial nitric oxide synthase (eNOS; rabbit polyclonal, RR-1711-R7, Neomarkers; Lab Vision, Fremont, CA, USA) were used. The sections were incubated with primary antisera (including CD31 or eNOS) for 1 h at room temperature. After washing in phosphate-buffered saline, the tissues were incubated with biotin-conjugated secondary antibody and then a streptavidin-biotin system for 30 min at room temperature. The reactions were visualized using diaminobenzidine tetrahydrochloride. The sections were counterstained using hematoxylin, then cleared and mounted.

### Controls and grading of the immunostaining

Appropriate positive (placenta, capillary endothelium for CD31 and eNOS) and negative (omitted primary antibody) controls were evaluated simultaneously in all cases. All cytoplasmic staining was recorded as positive for eNOS and CD31. The extent and intensity of eNOS reactions were semi-quantitatively evaluated using a four-level grading system. Grade 0 was no apparent reaction product. Focal and minimal staining intensity was graded 1, and the most prominent staining reaction covering nearly the whole area of the specimen was classified as 3. Grade 2 was intermediate between 1 and 3.

### Statistical analysis

Statistical analyses were carried out using the SPSS software (v. 11.0 for Windows; SPSS Inc.; Chicago, IL). All values are expressed as means ± SD. *P-*values less than 0.05 were deemed to be statistically significant. Group comparisons were made by one-way analysis of variance (Kruskall-Wallis) followed, in cases of significance, by the Mann-Whitney U test.

## Results

Bleeding did not stop in four of the Group 2 animals and in three of the Group 4 animals, for a total of seven in the plain gauze tampon groups. The ABS-soaked gauze tampon stopped bleeding in all Group-1 and -3 animals.

The mean bleeding time in 15 animals with the gauze tampon in Groups 2 and 4 was 4.9 ± 0.6 s, and in 22 animals in the ABS-soaked tampon Groups 1 and 3 was 3.1 ± 0.6 s. The mean bleeding time in the ABS-applied groups was 36.7% shorter than that of the plain gauze tampon groups, producing a significantly shorter duration of bleeding in the ABS groups (*p *= 0.0001).

Distal aortic occlusion was not observed on Days 1 or 7 after the operation in any group.

Comparisons of the histopathological changes in Group 1 and 2 animals are depicted in Table 1. Necrosis was absent, and the intensity of the inflammatory reaction together with endothelial cell loss did not differ significantly between the groups.

**Table 1:** Light microscopical and immunohistochemical findings in the rats abdominal aorta on the operation day

	Group I (ABS)	Group II (Plain gauze)	P
**Histopathological changes**			
**Inflammatory reaction**	1.0 ± 0.5	1.0 ± 0.6	0.7
**Necrosis**	none	none	N/A
**Endothelial cell loss**	1.0 ± 0.3	1.1 ± 0.4	0.5
**Fibrin plug formation/Erythrocyte aggregation**	1.5 ± 0.6	0.18 ± 0.4	0.0001
**Immunohistochemical changes**			
**e-NOS staining**	1.9 ± 0.7	1.1 ± 0.4	0.0001

**Figure 1:**
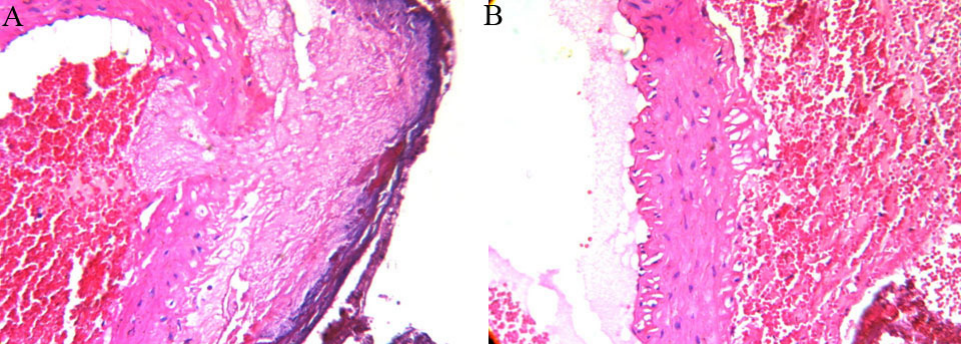
**Fibrin plug formation and erythrocyte aggregation at the arteriotomy site were more prominent in Group 1 (A) than in Group 2 (B)**. In the ABS tampon groups, microscopically evident basophilic discoloration in perivascular tissue was observed (A) (H&E, ×400).

Fibrin plug formation and erythrocyte aggregation at the arteriotomy site were more prominent in Group 1 than in Group 2. In the ABS tampon groups, a microscopically evident basophilic discoloration in the perivascular tissue was observed (Figure 1).

**Figure 2:**
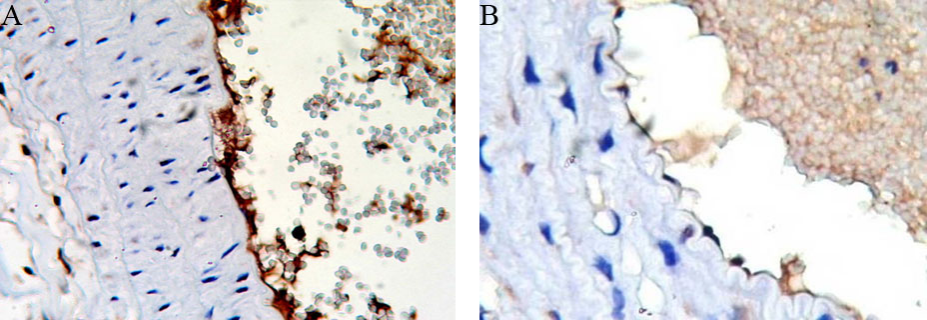
**A significantly more widespread and dense eNOS staining was observed in Group 1 (A) animals compared with Group 2 (B) (Immunohistochemistry, eNOS, ×400)**.

Significantly more widespread and dense eNOS staining was observed in Group 1 animals than Group 2 (Figure 2). The immunostaining of the unaffected vascular segments in ABS tampon and plain gauze tampon groups did not differ significantly for eNOS expression (Table 1). Comparisons of the histopathological changes on Day 7 of the operation are depicted in Table 2. There was no necrosis adjacent to the intimal and endothelial regeneration in either group.

**Table 2:** Light microscopical and immunohistochemical findings in the rats abdominal aorta on the 7^th ^day of the operation

	Group III (ABS)	Group IV (Plain gauze)	P
**Histopathological changes**			
**Necrosis**	none	none	N/A
**Endothelial cell egeneration**	all	all	N/A
**Foreign body reaction**	2.0 ± 0.7	1.1 ± 0.4	0.006
**Hemosiderin loaded hystiocyte**	1.8 ± 0.6	0.8 ± 0.6	0.001
**Immunohistochemical changes**			
**e-NOS staining**	0.8 ± 0.4	0.7 ± 0.4	0.6

**Figure 3:**
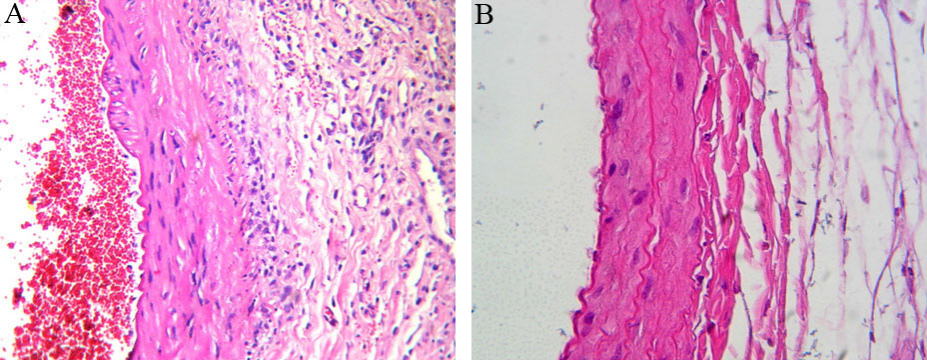
**Foreign body reaction on Day 7 after the operation in Groups 1 (A) and 2 (B) (H&E, ×400)**.

**Figure 4:**
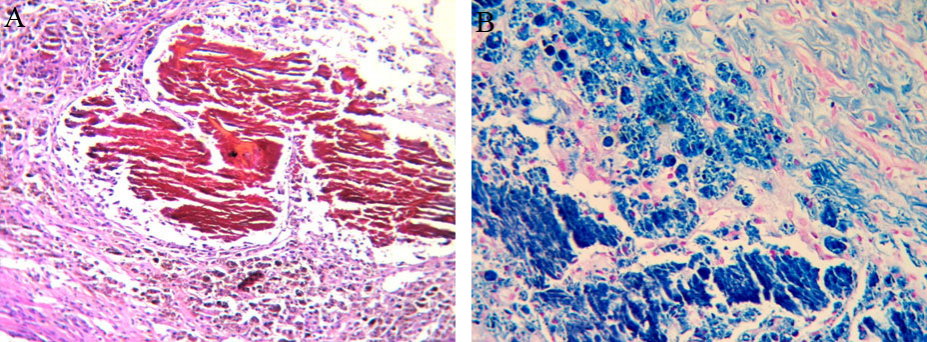
**In the ABS tampon groups, prominent hemosiderin-loaded histiocyte accumulation in perivascular tissue was observed (A-B) (A; H&E, B; iron blue, ×400)**.

The microscopically evident basophilic discoloration in the ABS group on the operation day was a result of a foreign body reaction (Figure 3A-B) and hemosiderin-loaded histiocyte accumulation on Day 7 after the operation (Figure 4A-B). Immunostaining with CD-31 showed an intact endothelial cell lining, and eNOS staining did not differ among groups on Day 7 after the operation.

## Discussion

Hemorrhage from anastomosis sites can usually be managed by additional sutures or light pressure. If adequate hemostasis cannot be achieved, various hemostatic agents may be used. The ideal hemostatic agent should be easy to use, require minimal training, show an effect within minutes, be effective in both arterial and venous bleeding, be non-toxic, and be anaphylactic [7]. Currently, no hemostatic agent possesses all of these characteristics.

ABS is a novel topical hemostatic agent that consists of various folkloric medicinal plant extracts (*Thymus vulgaris *0.1 mg, *Vitis vivifera *0.16 mg, *Glycyrrhiza glabra *0.18 mg, *Alpina officinarum *0.14 mg, and *Urtica dioica *0.12 mg). Each of these plants has vascular actions and some effect on the hematological system. *T. vulgaris *has anti-oxidative effects, such as prevention of lipid peroxidation [8]. *V. vivifera *has anti-atherosclerotic effects [9]. *G. glabra *decreases vascular endothelial growth factor production and cytokine-induced neovascularization [10]. *A. officinarum *inhibits nitric oxide production [11].

The hemostatic mechanism of ABS is effected by fibrinogen-erythrocyte agglutination, resulting in the formation of an encapsulated protein network that stimulates erythrocyte aggregation. This encapsulated protein network occurs very rapidly, in less than 1 s [12]. The ABS network might cover the entire physiological hemostatic process without affecting any individual clotting factor. Göker at al. demonstrated that coagulation factors II, V, VII, VIII, IX, X, XI, and XIII were not affected, and that plasma fibrinogen activity as well as total protein, albumin, and globulin levels were decreased by the addition of ABS to plasma [6,7]. These results showed that normal hemostatic elements were spared during the formation of the protein network. Thus, ABS might be useful in patients with antithrombotic drug-induced primary or secondary hemostatic abnormalities [13,14]. Cipil et al. demonstrated that ABS also had hemostatic effects in animals pretreated with warfarin. The bleeding time was reduced to 44% with ABS treatment [13].

Karakaya et al. demonstrated that ABS significantly reduced blood loss and death in experimental rat liver laceration [15]. Also, Dogan et al. used ABS for coronary artery bypass surgery patients. They sprayed 4-8 mL of ABS solution to bypass suture lines and the bleeding area. They indicated that patients who had used ABS required no revisions [16]. Our study revealed that fibrin plug formation and erythrocyte aggregation at the arteriotomy site were more prominent in Group 1 than in Group 2, and that bleeding time was 4.9 ± 0.6 s versus 3.1 ± 0.6 s in the ABS and control groups. Thus, ABS reduced bleeding time by 36.7% compared with the control group. In clinical experiments, ABS has been successfully used to control upper gastrointestinal bleeding [17,18], acute anterior epistaxis [19], and bleeding due to solitary rectal ulcers [20].

Although studies regarding the hemostatic effects and mechanism of ABS are available, there is no reported study regarding histopathological effects on vascular tissue. Negative effects of tissue topical agents used in anastomoses in cardiovascular surgery can influence the patency of grafts in both the short- and long-term.

Necrosis in vascular tissues, inflammatory reaction, and endothelial cell loss are important, particularly in terms of graft patency. Intimal hyperplasia can cause aneurysm and thrombus formation [1]. In our study, on Days 1 and 7 post-ABS application, histopathological changes in the rat abdominal aorta did not differ between Group 1 and 2 with regard to necrosis, inflammatory reaction, or endothelial cell loss.

After application of ABS, brown-colored changes occurred around the tissue [21]. We believe that the encapsulated protein network caused these changes. In the ABS tampon groups, a microscopically evident basophilic discoloration in the perivascular tissue was observed on the operation day and was caused by foreign body reaction and hemosiderin-loaded histiocyte accumulation. This status could be explained by the formation of the encapsulated protein network, causing delayed degradation of erythrocytes. The long-term clinical outcomes of this reaction must be clarified in prospective experimental studies.

*U. dioica*, one of medicinal plant extracts in ABS, causes vasodilatation by inducing nitric oxide production by the endothelium [22]. Significantly more widespread and dense eNOS staining was observed in Group 1 animals compared with Group 2. An increased eNOS level around arteriotomy areas in the early stages consistently stopped the bleeding *in vitro *without impairing tissue oxygenation or microcirculation of ABS.

The advantages of ABS when compared with other products that are readily available include effectiveness, ease of application, and no requirement for technical skills. However, as the product is relatively new, a limited amount of data is available related to long-term side effects and toxicity [1,21].

A limitation of this study is that only acute and early-stage effects of ABS were evaluated. Long-term anastomosis patency effects must be evaluated in further studies. Additional studies are required regarding possible effects of ABS on vascular tissues over a period longer than 7 days.

## Conclusions

This is the first reported study evaluating the histopathological and immunohistochemical effects of ABS on vascular structure. In this study, hemostasis was successfully achieved using ABS on rat abdominal aortas. No histopathological change in rat abdominal aortas between ABS and control groups on Days 1 and 7 was found. Further prospective studies are also required regarding long-term effects of foreign body reaction and hemosiderin-loaded histiocyte accumulation.

## Competing interests

The authors declare that they have no competing interests.

## Authors' contributions

OK: Acquisition, analysis and interpretation of data, surgical procedure, drafting of manuscript. MB, SAT: study design. NOK, AEG: performed microscopic and immunohistochemical evaluation and drafted the manuscript. EA: drafting of manuscript, design of the study. SG: interpretation of data, surgical procedure.

All authors have read and approved the final manuscript.
